# The Payment of Hospital Staffs

**Published:** 1910-09-10

**Authors:** G. A. Heron


*To the Editor of* The Hospital.

### THE PAYMENT OF HOSPITAL STAFFS.

*To the Editor of* The Hospital.

Sir,—Mr. Sydney Holland says, in effect, that medical men who do unpaid work in hospitals get what he calls a *quid pro quo* for that unpaid work in the prestige it gives them with their profession and in the remunerative practice it helps to bring to them.

I say, in effect, that the majority of medical men who give many days of the best years of their lives to unpaid hospital work get for it no *quid pro quo* of the kind Mr. Sydney Holland implies they receive.

I have expressed the opinion that, in the circumstances, Mr. Sydney Holland's statement is insulting to honorary medical officers of hospitals, and unworthy of one in the position he fills in the hospital world. That is, I hope, a clear statement of the only questions now at issue in your columns between Mr. Sydney Holland and myself. It is for him, if he so pleases, to make good his very remarkable estimate of the worth of the unpaid work done by medical men in hospitals.

Meanwhile, I am content to leave my dispute with Mr. Sydney Holland to the judgment—silent or otherwise—of those medical men who are competent to express an authoritative opinion on the subject.

Yours faithfully,

August 29, 1910. G. A. Heron.

